# Gaucher’s Disease, an Unusual Cause of Massive Splenomegaly, a Case Report

**Published:** 2013-10-22

**Authors:** F Binesh, A Yousefi, M Ordooei, MA Bagherinasab

**Affiliations:** 1 Associate Professor Of Pathology,Shahid Sadoughi University Of Medical Sciences,Yazd,Iran.; 2 Assistant Professor Of Pediateric Disease, Shahid Sadoughi University Of Medical Sciences,Yazd,Iran.; 3 General practitioner, Shahid Sadoughi University Of Medical Sciences,Yazd,Iran.

**Keywords:** Gaucher Disease, Splenomegaly, Diagnosis

## Abstract

**Background:**

Gaucher’s Disease (G.D.) is an autosomal recessive disorder resulting from the accumulation of glucocerebrosidase in the cells of macrophage-monocyte system as a result of a deficiency in lysosomal glucocerebrosidase. This enzyme is encoded by a gene on chromosome-1. Here we report a case of Gaucher’s Disease .G.D is rare in Yazd.

**Case reports:**

We reported a patient that presented with weakness, pallor and gradually increasing abdominal girth. Clinical examination and history pointed to be a lipid storage disease. Final diagnosis of G.D. was reported after examining the bone marrow smears. Confirmation of diagnosis on Gaucher’s disease was performed by measurement of glucocerebrosidase level.

**Conclusion:**

We report a case of G.D. to emphasize the importance of early recognition by clinical manifestation and histological findings. G.D. should be considered in the differential diagnosis of children with unexplained splenomegaly.

## Introduction

Gaucher disease (G.D.) is a lipid storage disease characterized by deposition of glucocerebroside in cells of the macrophage-monocyte system. It was first described by Gaucher in 1882, and the storage of glucocerebroside was first recognized by Epstein in 1924. The metabolic defect, which is the deficiency of the lysosomal hydrolase β-glucosidase, or β-glucocerebrosoidse, was identified by Brady et al (1). There are three clinical subtypes, which are delineated by the absence or presence and progression of neurologic involvement: type 1 or the non-neuronopathic form; type 2, the infantile-onset, acute neuronopathic form; and type 3, the juvenile-onset neuronopathic form (2). All three subtypes are inherited as autosomal recessive traits. Type 1 disease is the most common lysosomal storage disease and one of the most prevalent genetic disorders among Ashkenazi Jewish individuals. Its birth incidence in Ashkenazi Jewish is about 1 in 450 (3).

Here we report a case of GD presented with massive splenomegaly and cytopenia as the prodromal symptoms. Despite its rarity in Yazd, we presented this case to emphasize the importance of clinical examination and bone marrow finding in the diagnosis of G.D. Early diagnosis is important, because the disease is rare and diagnosis may be delayed. 

## Case report

A 3 year- old boy presented with weakness, pallor and gradually increasing abdominal girth. He was Muslim and born with a parents with no consanguineous marriage. He was delivered after full-term normal pregnancy. Development of the child was normal. The patient’s medical history was not significant. There was no history of easy bruising or prolonged bleeding on trauma, hematemesis, fever, night sweats, and weight loss or bone pains. 

**Figure1:**
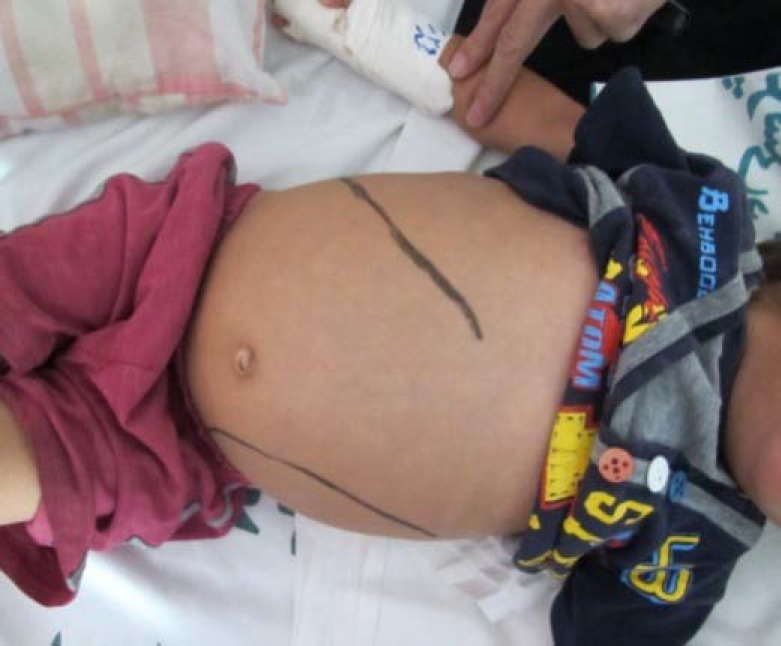
Massive splenomegaly is evident (case-1).

**Figure 2:**
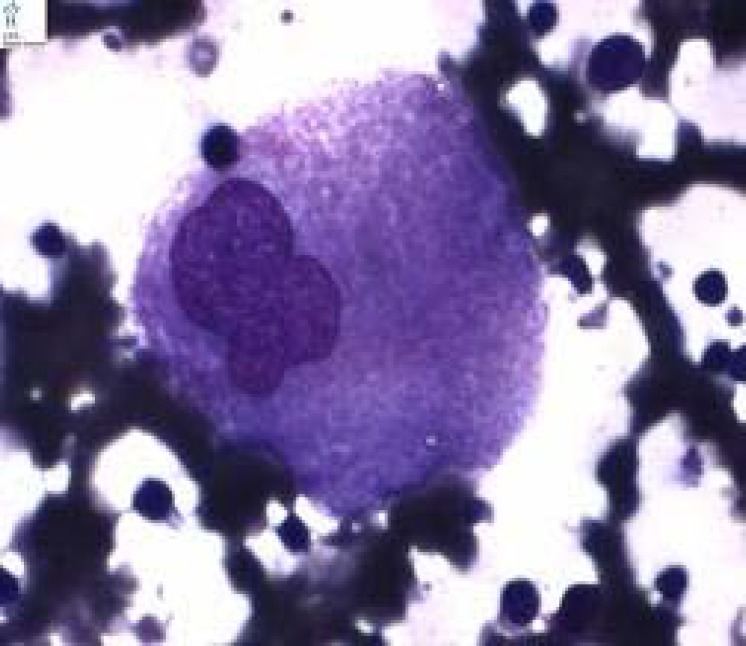
Gaucher cell in marrow smear (Wright Giemsa stain X100).

His family members, including his parents and two siblings, who were normal. Upon admission, the patient looked thin but not ill; he did not have fever. On physical examination, pallor was noted; however there was no icterus or lymphadenopathy. He had firm, non tender massive splenomegaly and a non tender, mild hepatomegaly (Figure 1). There were no signs of ocular motor problems or other neurological abnormalities. Rest of systemic examination was essentially normal. Lab investigations revealed bicytopenia (hemoglobin=8.5 g/dl, white blood cells=6.9x10^9^/L and platelets=114x10^9^/L). Liver enzymes were increased slightly to moderately (aspartate aminotransferase= 113IU/ml, alanine aminotransferase=61 IU/ml) but serum proteins and albumin, kidney function test and urine analysis were unremarkable.PT(prothrombin time) was 14_s_[ international normalized ratio(INR)=1.3] and PTT(partial thromboplastin time) was 42s.Mantoux(tuberculin sensitivity test or PPD test) viral markers, ANA (antinuclear antibody) and HIV (human immunodeficiency virus) antibody test were negative. Blood and urine cultures were negative. X-ray of the pelvis with lower limbs was found to be normal. Fundoscopy examination and hemoglobin electrophoresis were found to be normal. Ultrasound revealed grossly enlarged spleen (120x47mm) but splenic and portal veins had normal diameter. Liver span was 98mm with normal echotexture. To evaluate massive splenomegaly, bone marrow aspiration was performed which revealed Gaucher cells in a background of normal erythroid, myeloid and megakaryocytic lineage cells (Figure 2**).** Confirmation of diagnosis on Gaucher’s disease (type 1) was performed by βglucosidase levels-3.0 nmol/hv/mg (normal levels >6.0). Final diagnosis was G.D (type-1).

## Discussion

Gaucher’s disease is an autosomal recessive disorder .Its overall incidence is approximately 1:40,000 individuals (4) .It affects all racial and ethnic groups but prevalence is higher among Ashkenazi Jews. It is the most common lysosomal storage disorder (5). Clinical research shows that Gaucher disease manifests with broad phenotypic variation typical of many metabolic disorders, ranging from neonatal lethality to asymptomatic octogenarians. It has long been known that neither the amount of lipid stored, nor the residual enzymatic activity detected, correlates well with symptom severity (6). Although Gaucher's disease is well known in adult patients but about two-thirds of the patients present before the age of 20 and onset in childhood is predictive of severe and progressive phenotype(7). 

The most common signs and symptoms noted in GD are splenomegaly (95%), hepatomegaly (87%), radiological bone disease (81 %), thrombocytopenia (50%), anemia (40%), growth retardation (34%), bone pain (27%), and bone crisis (9%). A skeletal manifestation is found more often in older children (8) .International Collaborative Gaucher Group reported that 63% of patients have experience with bone pain and 26% develop bone crisis (9).The current case presented with organomegaly, failure to thrive and there was no evidence of bone disease even radiologically. In our case the differential diagnosis process began by considering the broad categories of disease that presented with hepatosplenomegaly: anatomical abnormalities, congestion, infection, hematologic disorders, and infiltrative processes .Although hematologic disorders could explain the organomegaly, several of these (including chronic hemolytic anemia, disorders associated with extramedullary hematopoiesis, myeloproliferative disorders, and sickle-cell disease) were ruled out because of the absence of other key signs such as jaundice, abnormal hemoglobin electrophoresis, painful crisis and leukocytosis. Within the remaining category of infiltrative processes, we considered two areas: malignant neoplasms and histiocytic disorders. Among the former, several childhood cancers could explain hepatosplenomegaly including leukemia, lymphoma and primary splenic tumors. However, most of these cancers present acute, rapidly developing symptoms, unlike the slow progression course in our case. In addition, the patient lacked other characteristic symptoms of such cancers, including appearance of illness, fever, chills and weight loss. Other types of cancer were deemed unlikely for the patient's age .Finally some histiocytic disorders could explain the patient's symptoms, but these were ruled out as unlikely for the patient's age and because he lacked other associated symptoms, such as rapid clinical deterioration, fever, wasting, skin rash and irritability. Among other histiocytic disorders, several metabolic storage disorders commonly present with hepatosplenomegaly.Gaucher disease was unique in its consistent with our patien's symptoms. B.M. examination is the hallmark for the diagnosis of G.D however; all suspects should be confirmed by demonstrating deficient acid β-glucosidase activity in isolated leukocytes (10). As we know pseudo-Gaucher cells may be found in the marrow of some patients with CML, type II congenital dyserythropoietic anaemia, thalassemia, Hodgkin lymphoma, multiple myeloma and AIDS. 

These patients do not lack the capacity to catabolise glucocerbroside (11) but the great inflow of glucoside in phagocytic cells exceeds their normal capacity to break down this glycolipid. The presented case had not any evidence of the above mentioned disorders. 

## Conclusion

G.D. should be considered in the differential diagnosis of patients with unexplained splenomegaly especially with an extended period of time. Moreover, the early recognition of GD would lead to safe and effective treatment with enzyme replacement which can decrease morbidity and reduce as far as possible the visceral and skeletal involvement.
